# Functional Lung MRI in Chronic Obstructive Pulmonary Disease: Comparison of T1 Mapping, Oxygen-Enhanced T1 Mapping and Dynamic Contrast Enhanced Perfusion

**DOI:** 10.1371/journal.pone.0121520

**Published:** 2015-03-30

**Authors:** Bertram J. Jobst, Simon M. F. Triphan, Oliver Sedlaczek, Angela Anjorin, Hans Ulrich Kauczor, Jürgen Biederer, Julia Ley-Zaporozhan, Sebastian Ley, Mark O. Wielpütz

**Affiliations:** 1 Department of Diagnostic & Interventional Radiology, University Hospital of Heidelberg, Heidelberg, Germany; 2 Translational Lung Research Center Heidelberg (TLRC), Member of the German Lung Research Center (DZL), Heidelberg, Germany; 3 Department of Diagnostic and Interventional Radiology with Nuclear Medicine, Thoraxklinik at University of Heidelberg, Heidelberg, Germany; 4 Department of Diagnostic & Interventional Radiology, Surgical Hospital Dr. Rinecker, Munich, Germany; 5 Research Center Magnetic Resonance Bavaria (MRB), Würzburg, Germany; 6 Radiologie Darmstadt, Department of Radiology Hospital Gross-Gerau, Gross-Gerau, Germany; 7 Institute for Clinical Radiology, Ludwig-Maximilians-University Hospital Munich, Munich, Germany; Clinica Universidad de Navarra, SPAIN

## Abstract

**Purpose:**

Monitoring of regional lung function in interventional COPD trials requires alternative endpoints beyond global parameters such as FEV1. T1 relaxation times of the lung might allow to draw conclusions on tissue composition, blood volume and oxygen fraction. The aim of this study was to evaluate the potential value of lung Magnetic resonance imaging (MRI) with native and oxygen-enhanced T1 mapping for the assessment of COPD patients in comparison with contrast enhanced perfusion MRI.

**Materials and Methods:**

20 COPD patients (GOLD I-IV) underwent a coronal 2-dimensional inversion recovery snapshot flash sequence (8 slices/lung) at room air and during inhalation of pure oxygen, as well as dynamic contrast-enhanced first-pass perfusion imaging. Regional distribution of T1 at room air (T1), oxygen-induced T1 shortening (ΔT1) and peak enhancement were rated by 2 chest radiologists in consensus using a semi-quantitative 3-point scale in a zone-based approach.

**Results:**

Abnormal T1 and ΔT1 were highly prevalent in the patient cohort. T1 and ΔT1 correlated positively with perfusion abnormalities (r = 0.81 and r = 0.80; p&0.001), and with each other (r = 0.80; p<0.001). In GOLD stages I and II ΔT1 was normal in 16/29 lung zones with mildly abnormal perfusion (15/16 with abnormal T1). The extent of T1 (r = 0.45; p<0.05), ΔT1 (r = 0.52; p<0.05) and perfusion abnormalities (r = 0.52; p<0.05) showed a moderate correlation with GOLD stage.

**Conclusion:**

Native and oxygen-enhanced T1 mapping correlated with lung perfusion deficits and severity of COPD. Under the assumption that T1 at room air correlates with the regional pulmonary blood pool and that oxygen-enhanced T1 reflects lung ventilation, both techniques in combination are principally suitable to characterize ventilation-perfusion imbalance. This appears valuable for the assessment of regional lung characteristics in COPD trials without administration of i.v. contrast.

## Introduction

Acquired chronic obstructive pulmonary disease (COPD) [1] is widely recognized as a clinically heterogeneous disease, and efforts are taken to identify and attribute key elements of the COPD syndrome to clinically meaningful subgroups (phenotypes), which can guide therapy more effectively [2,3]. Clinical parameters and pulmonary function testing (PFT) are currently used for severity assessment and therapy stratification [1], but they provide limited information to differentiate between different clinical subtypes [2,4]. In the last few years, computed tomography (CT) has become the method of choice for the regional assessment of the extent and distribution of structural lung alterations, such as emphysema, gas trapping and airway wall remodeling [3,4]. However, repeated therapy monitoring in short intervals would entail increased radiation exposure [5]. Proton magnetic resonance imaging (MRI) of the lung provides both regional structural as well as functional information without radiation exposure [6]. So far, dynamic contrast-enhanced (DCE) perfusion imaging is considered the most robust MR technology for obstructive airway disease [7,8]. It reflects the effect of hypoxic pulmonary vasoconstriction, meaning that lung perfusion images may be regarded as a surrogate for lung ventilation [9]. In COPD it has been shown to correlate with the degree of emphysematous destruction as quantified with CT [10]. Obviously, increased distal airspace due to hyperinflation, emphysema and hypoxic vasoconstriction in COPD result in a reduction of protons per volume. Since DCE MRI visualizes these pathologies indirectly and may fail to differentiate between perfusion deficits due to airway obstruction or emphysematous tissue loss, additional parameters for further characterization of lung tissue are wanted. A novel promising technique is the mapping of T1 relaxation times. T1 time is a physical parameter that directly correlates with composition and state of lung tissue, including the blood volume fraction [11]. Preliminary results indicate that T1 relaxation time is significantly shorter in lungs affected by emphysema compared to lungs of healthy subjects [12] or cystic fibrosis patients [13], but until today insufficient data on T1 relaxation time behavior in COPD are available [14]. It can be assumed, that T1 mapping can provide important additional information for the regional characterization of lung tissue. Moreover, as a physical parameter, it might even provide an objective parameter for the characterization of pulmonary diseases independent of scanner type or observer [13]. Furthermore, T1 mapping can be combined with oxygen-enhanced (OE) MRI which exploits the paramagnetic effect of molecular oxygen (O_2_). Elevated oxygen levels lead to a measurable T1 time shortening in ventilated lungs due to dissolved O_2_ [15]. This is the basis of using OE MRI to display pulmonary ventilation, by monitoring either the T1-weighted signal increase between images acquired breathing room air and after supplementing O_2_, or mapping T1 relaxation time itself. In a previous study, Ohno et al. could already demonstrate that the O_2_-induced T1 signal increase was reduced in patients with emphysema and correlated with PFT and pulmonary diffusion capacity [16]. With this background, it was the aim of the present study to explore the potential value of T1 mapping and oxygen enhanced T1 mapping as a new imaging techniques for the comprehensive assessment of regional lung disease in COPD. Thus, we semi-quantitatively assessed T1 maps and oxygen enhanced T1 maps, and correlated the results with DCE first-pass perfusion imaging and disease severity in 20 patients with COPD of different severity to assess the predictive value of the above mentioned T1 mapping techniques for the pulmonary blood pool and ventilation/perfusion imbalance.

## Materials and Methods

### Patient characteristics

20 patients diagnosed with clinically stable COPD according to the GOLD criteria [17] were enrolled into a prospective study, in particular 10 patients with GOLD stages I-II and 10 patients with GOLD stages III-IV (Table 1). Subjects were predominantly male (17/20) and their ages ranged from 49 to 79 years (median age 66 years) (Table 1). Spirometry was available for every patient, performed according to the guidelines of the European Respiratory Society and the standards of the American Thoracic Society [18], with the European Coal and Steel Community predicted values serving as the standard of reference [19].

**Table 1:** Summary of patient characteristics.

	Mild COPD		Severe COPD		
	GOLD1	GOLD2	GOLD3	GOLD4	Overall
**n**	2	8	3	7	20
**Male (n)/ Female (n)**	2/0	6/2	3/0	6/1	17/3
**Age (y)**	54 (50–57)	61 (49–77)	76 (71–77)	67 (60–79)	66 (49–79)

Data are median and range in brackets

### Ethics statement

The study was approved by the Institutional Review Board of the Medical Faculty of the University of Heidelberg, Germany. All subjects gave written informed consent for examination and data evaluation. The work was carried out in accordance with The Code of Ethics of the World Medical Association (Declaration of Helsinki).

### Magnetic Resonance Imaging

All examinations were performed on a clinical 1.5 T scanner (Magnetom Avanto, Siemens Medical Solutions, Erlangen, Germany). First, T1 mapping of the whole lung was performed breathing room air (T1) using an Inversion Recovery (IR) snapshot FLASH sequence [20] in coronal orientation in expiration at 8 positions in each lung with a breath-hold time of 6 seconds per slice as previously described [13,21]. The individual images were acquired with a short echo time of TE = 750 μs using an asymmetric readout to compensate for the short T2* in lung tissue. With TR = 3ms, each image acquisition took 192 ms, giving sufficient time to sample the inversion recovery. MR excitation was performed at a flip angle of 8° to reach the effective Ernst angle in the lung area for a T1≈1100 ms. Following a global inversion pulse, a series of 32 FLASH images were acquired and T1 maps calculated using a per-pixel fit from the effective T1 relaxation exhibited in this series. The T1 fit was implemented in the image reconstruction framework of the scanner. The resulting spatial resolution of the T1 maps was identical to the originally acquired images with 3.9 × 7.8 mm^2^ in-plane and 15 mm slice thickness. After these first measurements, a clinical oxygen mask was positioned over the patient´s nose and mouth, without moving the patient, and pure O_2_ was supplemented at 15 l/min to ensure a steady state of pulmonary hyperoxia. In this context, previous animal studies demonstrated a linear decrease increase of pulmonary parenchymal relaxivity during the first 2 minutes of oxygen supplementation [22]. Within approximately 3 minutes of ventilation with pure oxygen, a steady state of pulmonary hyperoxia automatically occurs in human subjects. The resulting T1 reduction of 8–10% relative to baseline T1 remains constant during steady state of pulmonary hyperoxia with low inter-subject variability [23]. Therefore, the acquisition of T1 maps was performed during ventilation with molecular oxygen at bespoke steady state of hyperoxia and was started with a delay of 5 minutes in every patient to ensure standardized image acquisition. In this process, the identical IR FLASH sequence was repeated at identical positions, and oxygen enhanced T1 maps (T1O_2_) were computed. 5 minutes after terminating hyperoxia, we performed DCE perfusion imaging with a commercially available three-dimensional gradient echo (GRE) T1-weighted sequence pitched to high temporal resolution (TWIST) (1.5 s per volume) during inspiratory breath-hold and contrast injection by a power injector (0.05 mmol/kg body weight Gd-DTPA, Magnevist, Bayer Schering Pharma AG, Berlin, Germany) at a rate of 5 ml/s followed by a saline chaser of 30 ml injected at a rate of 5 ml/s with 30 consecutive measurements and a spatial resolution of 2.0 x 2.0 mm in-plane and 5 mm slice thickness. In-room time for the whole procedure approximated 30 minutes per patient.

### Data analysis

T1 and T1O_2_ were translated into color-encoded parameter maps. We applied a color scale optimized for highlighting T1 variations in the pulmonary parenchyma, which covered a T1 range from 1 ms (dark blue) to 1800 ms (dark red). Voxels with insufficient signal for a T1 fit were displayed as black and voxels above the threshold of 1800 ms were displayed as white. From DCE datasets, greyscale perfusion images were generated by subtracting pre-contrast acquisitions from the time point with highest enhancement of lung tissue, thus depicting pulmonary blood volume as previously described[24].

All 20 MRI datasets of COPD patients were reviewed in consensus by 2 chest radiologists with 2–4 years of experience specifically in lung MRI and evaluated according to the following semiquantitative scoring sheet. The right and left lung were each divided into 3 zones: upper, middle, and lower zone, i.e. in 6 zones per patient. The spatial extent of each abnormality in each lung zone was rated with a semiquantitative 3-point Likert rating scale with 0 = normal; 1 = <50% of lung zone affected; 2 = >50% of lung zone affected. The following criteria were evaluated I) Areas with T1<1000ms. II) Lack of a significant color shift on T1O_2_ maps from dark red towards dark blue when compared to T1 (ΔT1 = T1—T1O_2_ < 50 ms), meaning an absence of oxygen-induced T1 reduction (ΔT1). Thus, a low baseline T1 occasionally prohibited the detection of ΔT1. III) Areas of perfusion abnormalities with reduced or missing peak enhancement on DCE subtraction data sets. Sum scores were calculated for every category by adding up scores of all 6 lung zones, thus scores could range from 0–12. Consensual reading averaged 15 min per case.

### Statistical analysis

Calculations were done using SigmaPlot 12.5 (Systat Software Inc., San Jose, CA, USA). The Spearman rank order correlation coefficient was calculated for T1 with ΔT1, perfusion abnormalities and GOLD stages. By convention, r between 0.0–0.2 was regarded as negligible, 0.2–0.4 as weak, 0.4–0.7 moderate, 0.7–0.9 strong, and 0.9–1.0 very strong correlation [25]. The closeness of agreement was assessed by Weighted Kappa and the following levels of agreement: 0–0.20 = poor, 0.21–0.40 = fair, 0.41–0.60 = moderate, 0.61–0.80 = substantial, 0.81–1.00 = almost perfect [26]. Multiple testing was compensated for by the Bonferroni-Holm method [27].

## Results

### Abnormal T1 is highly prevalent in COPD

The examination could be completed in 18 of 20 patients with diagnostic quality, while two patients refused intravenous contrast agent application. T1 abnormalities were found in 19/20 (95%) patients. In a per-zone analysis, decreased T1 was prevalent in 97 out of 120 lung zones (81%) (Table 2). After supplementing O_2_, abnormal ΔT1 was detected in a total of 17/20 (85%) patients and 84/120 (70%) lung zones showed abnormal ΔT1 (Table 2). 17/18 (94%) patients showed perfusion abnormalities, which were present in 87/108 (81%) of lung zones (Table 2). Abnormalities of T1, ΔT1 and perfusion were present simultaneously in the majority of patients (15/20; 75%). 2/20 (10%) patients showed abnormalities of T1 and perfusion, but normal ΔT1. A single patient (1/20; 5%) showed alterations neither of T1, ΔT1 nor perfusion.

**Table 2:** Comparative assessment of T1 mapping at room air, pure O_2_ and perfusion.

	Score = 0 n(%)	Score = 1 n(%)	Score = 2 n(%)	Total n(%)
**T1**	23 (19%)	56 (47%)	41 (34%)	120 (100)
**ΔT1**	36 (30%)	43 (36%)	41 (34%)	120 (100)
**Perfusion**	21 (19%)	44 (41%)	43 (40%)	108 (100)

Severity of abnormal T1 at room air (T1), abnormal T1 reduction with 100% O_2_ (ΔT1) and perfusion alterations (Perfusion) in a per-zone approach. Note that perfusion imaging was rejected by 2 patients.

### T1 abnormalities correlate with perfusion abnormalities

**Fig 1:**
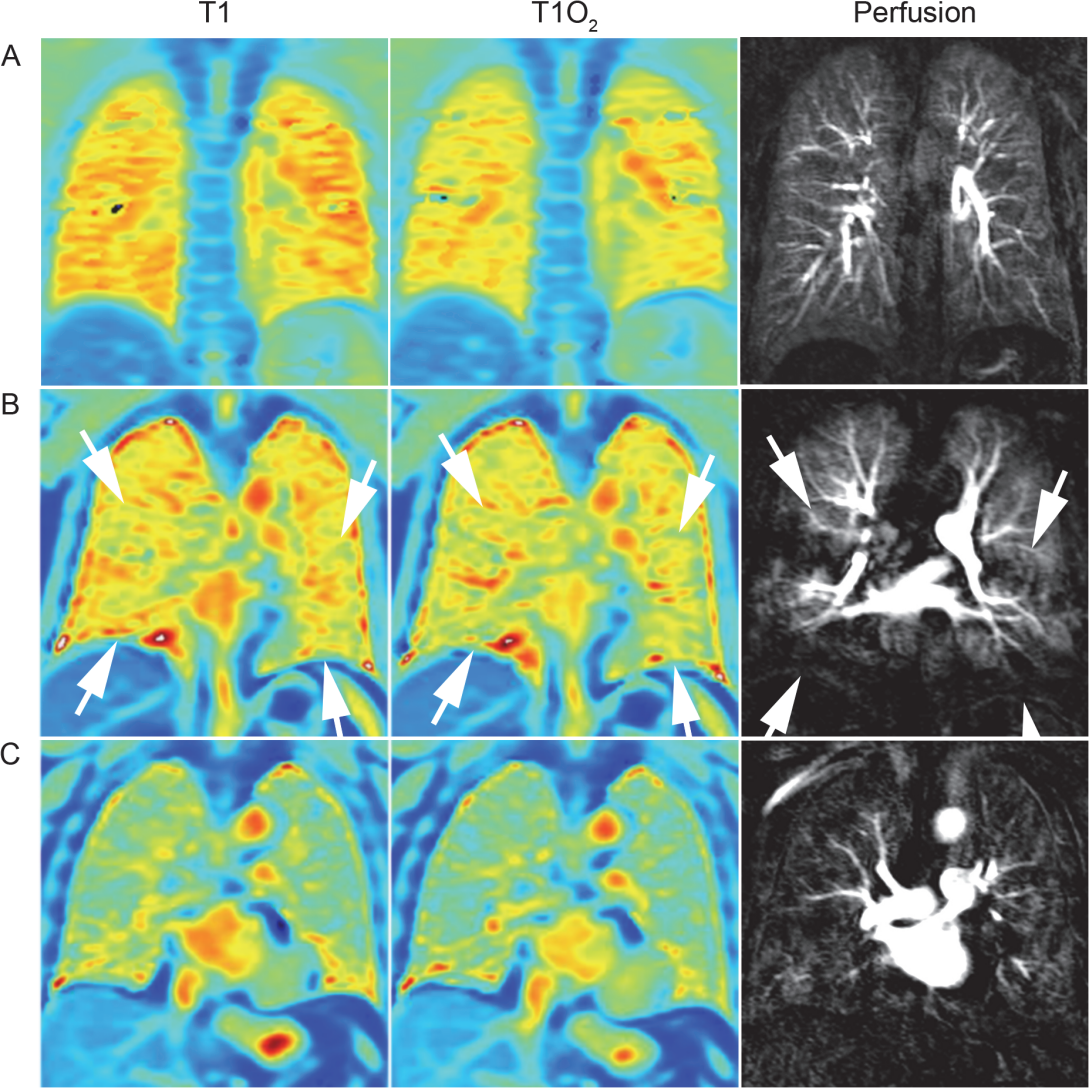
Comparison of T1 and perfusion characteristics. Normal T1 mapping at room air (left), T1 mapping after 100% O2 (middle), and DCE lung perfusion (right) (zonal scores = 0 each) of a 29-year-old healthy proband (A). 59-year-old patient with GOLD stage II showing minor T1 abnormalities at room air (score = 1), normal ΔT1 after 100% O2 (score = 0) and minor perfusion abnormalities (score = 1) of the middle and lower lung zones (area between white arrows), reflecting lung areas with intact ventilation and simultaneous perfusion impairment (B). Severe T1 abnormalities at room air, abnormal ΔT1 after 100% O2 and perfusion abnormalities (score = 2 each) affecting the entire lung of a 75 year old patient with GOLD stage II (C).

In a zone-based approach, baseline T1 abnormalities showed a strong correlation with perfusion abnormalities (r = 0.81; p<0.001) (Table 3, Fig. 1). Moreover, ΔT1 abnormalities correlated positively both with perfusion (r = 0.80; p<0.001) and T1 abnormalities (r = 0.80; p<0.001) (Table 3, Fig. 1). Weighted kappa indicated almost perfect agreement for T1 abnormalities and perfusion abnormalities (ĸ = 0.82; 95%CI(confidence interval) = 0.74–0.90). ΔT1 abnormalities and perfusion abnormalities showed substantial agreement (ĸ = 0.75; 95%CI = 0.68–0.83). Only 2/86 lung zones with T1 abnormalities and 3/73 lung zones with ΔT1 abnormalities showed normal perfusion (Table 4). At a zonal level, the differences between scores for T1, ΔT1, and perfusion never exceeded 1 score point.

**Table 3:** Cross-correlation of T1 reduction at room air, with pure O_2_ and perfusion.

COPD stage		Lung zones n	r	p
	T1 vs. Perfusion	108	0.81	<0.001
**Overall**	ΔT1 vs. Perfusion	108	0.80	<0.001
	ΔT1 vs. T1	120	0.80	<0.001
	T1 / Perfusion	54	0.85	<0.001
**Mild (GOLD I&II)**	ΔT1 / Perfusion	54	0.63	<0.001
	ΔT1 / T1	60	0.65	<0.001
	T1 / Perfusion	54	0.67	<0.001
**Severe (GOLD III&IV)**	ΔT1 / Perfusion	54	0.73	<0.001
	ΔT1 / T1	60	0.77	<0.001

Spearman rank order correlation coefficient r calculated for T1 abnormalities at room air (T1), abnormal T1 shortening with 100% O_2_ (ΔT1) and perfusion abnormalities (Perfusion), grouped according to COPD stages in a per-zone approach.

**Table 4:** Correlation table for abnormal T1 at room air (T1), T1 shortening with 100% O_2_ (ΔT1) and perfusion abnormalities, grouped according to COPD stages in a per-zone approach.

		GOLD I&II				GOLD III&IV			
		Perfusion				Perfusion			
		0	1	2	Sum	0	1	2	Sum
T1	0	**13**	2	0	15	**6**	1	0	7
	1	2	**26**	2	30	0	**11**	10	21
	2	0	1	**8**	9	0	3	**23**	26
	Sum	15	29	10	**54**	6	15	33	**54**
ΔT1	0	**12**	16	0	28	**6**	1	0	7
	1	3	**13**	2	18	0	**12**	9	21
	2	0	0	**8**	8	0	2	**24**	26
	Sum	15	29	10	**54**	6	15	33	**54**
		T1				T1			
		0	1	2	Sum	0	1	2	Sum
ΔT1	0	**12**	17	0	29	**7**	0	0	7
	1	4	**15**	1	20	0	**19**	4	23
	2	0	0	**11**	11	0	5	**25**	30
	Sum	16	32	12	**60**	7	24	29	**60**

### Abnormal T1 and oxygen-enhanced T1 correlate with lung function

We subsequently formed two groups with either mild (GOLD I&II) or severe COPD (GOLD III&IV). When looking at the score of individual lung zones (Table 4), there was a tendency to higher scores in patients with severe COPD compared to mild COPD. In mild COPD, deviations between ratings of T1 and perfusion imaging were observed only rarely, meaning an almost perfect agreement of abnormal T1 and abnormal perfusion (ĸ = 0.85; 95%CI = 0.75–0.96) (Table 4). In contrast, there was only a substantial agreement between ΔT1 and perfusion abnormalities in mild COPD (ĸ = 0.63; 95%CI = 0.47–0.79). This is mainly because in mild COPD, 16/29 lung zones with minor perfusion deficits (score = 1) showed normal ΔT1 (score = 0) (Table 4). This suggests that perfusion alterations were present without concomitant ventilation impairment. 15 of these 16 lung zones showed minor T1 abnormalities (score = 1) in consent with the perfusion study. Consequently, the level of agreement between ΔT1 restriction and T1 abnormalities was also substantial in mild COPD (ĸ = 0.67; 95%CI = 0.52–0.81).

In patients with severe COPD, the agreement of T1 and perfusion abnormalities tended to be lower (ĸ = 0.74; 95%CI = 0.58–0.89) than in mild COPD (Table 4), but the agreement of ΔT1 and perfusion alterations tended to be higher (ĸ = 0.77; 95%CI = 0.64–0.91) (Table 4). Interestingly, T1 scores were often lower than those of concomitant perfusion abnormalities. 10/33 lung zones showed minor alterations of T1 (score = 1), but severe perfusion deficits (score = 2) (Table 4), indicating that the extent of pulmonary abnormalities in severe COPD may be underestimated by T1 alone (Table 4). In particular, in 6 of these lung zones, T1 and ΔT1 restriction matched each other. Importantly, there was also a significant correlation between T1, ΔT1, as well as perfusion abnormalities and GOLD stage (r = 0.45, 0.52 and 0.52, respectively) (Table 5).

**Table 5:** Correlation of T1 mapping at room air and with 100% O_2_, and perfusion with COPD severity.

	Cases n	r	p
**GOLD vs. Perfusion**	18	0.52	<0.05
**GOLD vs. T1**	20	0.45	<0.05
**GOLD vs. ΔT1**	20	0.52	<0.05

Spearman rank order correlation coefficient r calculated for abnormal T1 at room air (T1), abnormal T1 shortening with pure O_2_ (ΔT1) and perfusion alterations (Perfusion), with COPD stage in a per-patient approach.

## Discussion

DCE MRI has already demonstrated the capability to detect perfusion abnormalities in patients affected by COPD-related emphysema [28] and cystic fibrosis [24]. In emphysema, perfusion impairment is the result of tissue destruction and subsequent loss of the capillary bed [29], in case of airway obstruction in COPD or cystic fibrosis it is the result of local hypoxic pulmonary vasoconstriction [9,30]. However, both mechanics occur in COPD and are closely linked to a different degree in each individual patient. In the present study, we tried to cross-correlate T1 mapping and oxygen-enhanced T1 mapping against perfusion MRI as a new method to study the regional pulmonary blood pool and regional ventilation/perfusion imbalance in COPD patients.

In summary, the spatial extent and distribution of T1 abnormalities strongly correlated with perfusion abnormalities in DCE MRI, especially in COPD stages I and II. In the healthy lung, the free water protons of the pulmonary blood pool are the primary source of T1 signal and outweigh the signal from water in the surrounding tissue, resulting in relatively long T1 relaxation times close to—but still significantly shorter than—the corresponding blood T1 [11,31,32]. In lung areas with reduced lung perfusion and thus less signal contribution from blood, pulmonary T1 more strongly depends on the pulmonary parenchymal proton fraction, which is assumed to have a shorter T1 [11,14]. This is supported by our data, as we observed areas with shorter baseline T1 which strongly matched perfusion abnormalities with regard to localization and spatial extent. Under the assumption that a shorter T1 reflects primarily reduced blood content per volume, our data indicate that T1 mapping at room air provides a surrogate marker for the pulmonary blood volume, especially in patients with GOLD stages I and II.

In patients with GOLD III and IV, the extent of T1 abnormalities at room air underestimated the size of perfusion abnormalities in several lung zones with large perfusion abnormalities. We speculate that this effect is partially related to the higher slice thickness of the T1 maps (15 mm) compared to perfusion images (5 mm), meaning that residual areas of normal blood content within the MR slice may outweigh areas of reduced blood fraction since the measured T1 is weighted by proton density. We assume that this did not come into effect in the patient group with mild COPD, as lung areas with large perfusion abnormalities were infrequent in these patients. Nevertheless, when considered in isolation, the presence or absence of abnormalities on T1 maps at room air and perfusion images showed a high agreement in both patient groups.

In this study, ΔT1 strongly correlated with perfusion abnormalities, as long as an initially sufficiently high baseline T1 allowed for a ΔT1 > 50 ms. In healthy lungs, the inhalation of 100% molecular oxygen results in a T1 shortening of about 8–10% relative to baseline T1 at room air [23] due to the paramagnetic attributes of molecular oxygen. Since this reflects the presence of O_2_ both in tissue water and blood, it provides an indirect measure of ventilation, perfusion and oxygen diffusion through alveolar walls [11]. In COPD, a restriction of ΔT1 can be assumed to result either from reduced O_2_ inflow (impaired ventilation) or reduced perfusion [7], which are however closely linked in COPD-related gas trapping and emphysema. This is confirmed by our findings with a good agreement between oxygen-enhanced T1 mapping and DCE perfusion. Remarkably, we found that oxygen-enhanced T1 mapping was normal in few lung zones with minor baseline T1 and perfusion abnormalities in GOLD stages I and II. Presumably, these lung areas had intact ventilation and reduced but sufficient blood content per volume, resulting in a normal ΔT1 of more than 50 ms during ventilation with O_2_. This particular finding is of relevance, since the potential for therapeutic intervention might be especially large in early stage COPD, and further research is warranted to explore the diagnostic value of lung tissue characterization by T1 mapping. The T1 relaxation time at room air and during ventilation with O_2_ as an absolute parameter for the state of lung tissue could at least provide important additional information to increase the diagnostic value DCE lung perfusion imaging. Interestingly, similar findings were reported by Nakagawa et al. in patients with pulmonary embolism [33]. In their study, 6/6 patients with visible defects on MRI perfusion images were free of ventilation defects assessed by dynamic oxygen-enhanced MRI and ventilation scintigraphy [33]. However, we did not have clinical or radiological evidence for pulmonary embolism in our cohort of patients. A V/Q mismatch due to other causes than pulmonary embolism (i.e. increased V/Q ratio due to genuine perfusion impairment) was also reported by Rodriguez-Roisin et al., who demonstrated the presence of clearly abnormal ventilation-perfusion ratio already in early stages of COPD using multiple inert gas elimination technique [34].

Only a small number of lung zones with impaired ventilation on oxygen-enhanced T1 mapping showed normal perfusion, or larger ventilation impairments than perfusion deficits. We propose that these lung areas with reduced V/Q reflect right-to-left shunts, which was described by Wagner et al., who reported shunts greater than 5% of the cardiac output in as few as 2/23 COPD patients [35]. The frequency and spatial extent of signal alterations detected by T1 mapping, oxygen-enhanced T1 mapping and perfusion abnormalities increased from mild to severe COPD, and each showed a moderate agreement with GOLD stage. Despite using a different approach (dynamic oxygen-enhanced MRI delivering relative signal changes) in a prospective multi-center trial, Ohno et al. also reported a significant association between the mean relative enhancement ratio during supplementation of 100% oxygen and air flow limitation (FEV1%) in 160 smokers [36].

The present study was focused on the spatial extent and localization of functional lung changes in COPD by quantitative T1 mapping and quantitative oxygen-induced T1 reduction, as this approach promised to be robust, scanner-independent and standardized for potential clinical application. A similar assessment method has already been successfully applied in the scoring of morphological and functional MRI in patients with cystic fibrosis, rating the spatial extent of airway and parenchymal pathologies as well as perfusion deficits on a 3-point scale [24]. This approach provided information on the size and localization but not on the intensity of signal alterations. We favored a zonal approach over a lobe-based approach, because the lung fissures cannot be identified on T1 maps. Theoretically, regional T1 relaxation times and dynamic contrast enhanced perfusion parameters such as the maximum signal enhancement, the pulmonary blood flow, pulmonary blood volume, and mean transit time are amenable to software-based quantitative evaluation [37,38] which is likely to be more sensitive than visual scoring. But a valid quantitative comparison of these modalities would require software-based segmentation including elastic registration of volumetric perfusion data and 2-dimensional T1 maps (8 coronal slices per lung), which is prone to failure due to the differences in data architecture such as volumetric vs. discontinuous acquisition as well as differences in slice thickness. Additionally, image registration is complicated by the fact that T1 maps were acquired in expiratory breath hold as recommended previously [21], whereas perfusion images were acquired in inspiration. Currently, there is to our knowledge no post-processing software which would meet these requirements. Our static approach did not allow for a differentiation between areas of diminished or delayed ventilation, as it has been previously described in COPD by ^3^HE-MRI [39]. While dynamic oxygen-enhanced MRI methods may provide additional information, they require much longer measurement times and more complicated post-processing. Moreover, a recent study by Ohno et al. has revealed that dynamic O_2_ MRI parameters did not differentiate better between different stages of COPD than static O_2_ enhancement similar to the approach in our study [40]. As 3D sequences for T1 mapping are not yet available, we had to rely on 2D sequences acquiring 8 coronal slabs per lung, thus implying partial volume effects. In the future, 3D datasets together with matched morphological and perfusion sequences could allow for a complete mapping of the lungs with identical slice thickness and thus facilitate a quantitative analysis. The use of 3D datasets with identical slice thickness may prevent partial volume effects and thereby eliminate the underestimation of pulmonary blood volume reduction as we observed in some patients with large perfusion abnormalities. Moreover, further evaluation of T1 mapping techniques with MRI perfusion and quantitative CT data is necessary to determine the full potential of T1 mapping for the assessment of regional ventilation-perfusion imbalance and tissue destruction in COPD patients.

In conclusion, T1 mapping at room air correlated with perfusion deficits as shown in comparison with contrast enhanced dynamic MRI. Under the assumption that T1 reflects the regional pulmonary blood pool without using contrast medium, it might be of practical value especially in mild COPD. Furthermore, oxygen-enhanced T1 mapping reflects effects of lung ventilation. Together with dynamic contrast enhanced MRI perfusion it may contribute to the characterization of ventilation-perfusion mismatches. Since both T1-techniques correlated with disease severity and differentiate regional lung characteristics, they might be valuable for outcome assessment in COPD trials. Further research is warranted to explore the potential additional diagnostic value of T1 mapping alone or in combination with OE-MRI beyond the diagnostic scope of dynamic contrast enhanced perfusion MRI, which served as standard of reference in this preliminary study. Further development is warranted to refine the technology towards acquisition of volumetric data.
